# The zebrafish HGF receptor *met* controls migration of myogenic progenitor cells in appendicular development

**DOI:** 10.1371/journal.pone.0219259

**Published:** 2019-07-09

**Authors:** Hanna Nord, Nils Dennhag, Hanna Tydinger, Jonas von Hofsten

**Affiliations:** 1 Department of Integrative Medical Biology, Umeå University, Umeå, Sweden; 2 Umeå Centre for Molecular Medicine, Umeå University, Umeå, Sweden; Stanford University School of Medicine, UNITED STATES

## Abstract

The hepatocyte growth factor receptor C-met plays an important role in cellular migration, which is crucial for many developmental processes as well as for cancer cell metastasis. C-met has been linked to the development of mammalian appendicular muscle, which are derived from migrating muscle progenitor cells (MMPs) from within the somite. Mammalian limbs are homologous to the teleost pectoral and pelvic fins. In this study we used Crispr/Cas9 to mutate the zebrafish *met* gene and found that the MMP derived musculature of the paired appendages was severely affected. The mutation resulted in a reduced muscle fibre number, in particular in the pectoral abductor, and in a disturbed pectoral fin function. Other MMP derived muscles, such as the sternohyoid muscle and posterior hypaxial muscle were also affected in *met* mutants. This indicates that the role of *met* in MMP function and appendicular myogenesis is conserved within vertebrates.

## Introduction

Cellular migration is one of the corner stones of the processes that forms the structure and organisation of the multicellular organism and has important implications for development and disease. The migratory muscle progenitor cells (MMPs) are a group of cells which originally reside within the embryonic somite and upon inductive signals the MMPs delaminate from the somite and migrate laterally, giving rise to hypaxial muscle groups [1–3]. MMPs populate the limb buds and give rise to the limb musculature in mammals and fin musculature in fish [4] and at least in amniotes the MMPs also give rise to muscle in the tongue and the diaphragm [5–7].

The formation of muscle cells in mammals is initiated in the presomitic mesoderm where inductive signals, such as HH and FGF from the midline and surrounding embryonic structures initiates a myogenic programme in the somites and expression of the myogenic regulatory factors (MRFs, e.g Myf5, MyoD and Myogenin) results in the subsequent differentiation of muscle fibres. In the early stages of mammalian somitic compartmentalization, the transcription factor Pax3 is expressed throughout the mesodermal cells in the whole somite [8]. As the somite becomes divided into substructures with specialized fates the expression of Pax3 becomes restricted to the dermomyotome and concentrated to its epaxial and hypaxial lips, where it acts as an up-stream regulator of *Myf5* and *MyoD* expression [9, 10]. Pax3 also plays an important role in controlling the MMPs that move out from the somite to form muscle cells within the limbs [11, 12], in part by regulating the expression of the hepatocyte growth factor (HGF) receptor molecule *C-met* [13, 14]. C-met is essential for limb muscle development in amniotes, where the loss C-met results in severely impaired limb muscle [5, 6]. C-met and HGF mediate the epithelial-to-mesenchymal transition (EMT) of the delaminating MMP cells [15–17]. The transcription factor Lbx1 also play an important role in limb muscle precursor cells as blocked Lbx1 expression leads to unsuccessful MMP migration in mice [5, 18]. Lbx1 plays a similar role in zebrafish where FGF signalling from within the fin bud controls the Lbx1 phosphorylation state, which is important for MMP function in the zebrafish pectoral fin development [19]. The RAS-RAF pathway, particularly BRAF, positively regulates and directly activates Pax3 and subsequently C-met to drive MMP derived limb muscle formation [20]. The MMPs are also guided through the lateral mesoderm toward the limb bud by the chemokine receptor CXCR4 which enable the MMPs to be attracted to the ligand XCL12/SDF-1 expressing limb mesenchyme [21–23]. The patterning of the limb, including muscle within the limb, is subsequently organised by a handful of signalling centres and extrinsic signals [1, 24–27]. Forelimb or hindlimb patterning is determined by homeobox factor Pitx1 and the T-box factors tbx4 and Tbx5 [28, 29]. During the migration from the somite MMP cells will receive signals from the lateral plate mesenchyme and become divided into dorsal and ventral subclusters that will give rise to the abductor and adductor muscle respectively [30].

The similarities between the appendicular structures limbs and fins are many, including several of the genes that orchestrate their respective developmental processes. The zebrafish pectoral fins are homologous to amniote forelimbs and its muscles are, like in the amniote limbs, derived from MMPs with somitic origin [4, 31, 32]. In the zebrafish embryo, MMPs from the anteriormost somites migrate to the oesophagus, the sternohyoid muscle, the pectoral fins, and the posterior hypaxial muscle [31]. Later, during juvenile development, the pelvic fin musculature forms from MMPs deriving from somites 9–12 [33].

The zebrafish *C-met* orthologue *met* is expressed in various cell types during embryogenesis, including cells in the hypaxial myotome region and the developing pectoral fins [34]. Functional analyses of zebrafish *met* in myogenesis, using morpholino experiments, indicate that the lack of *met* leads to disturbed hypaxial and appendicular myogenesis, although *met* is not needed for the initial specification of these cells [35]. In this study, we use a genetic approach to study the role of the zebrafish *met* gene during the migration of MMPs in the formation of pelvic fin and pectoral fin musculature.

## Results

### Met is required for normal appendicular muscle development

**Fig 1:**
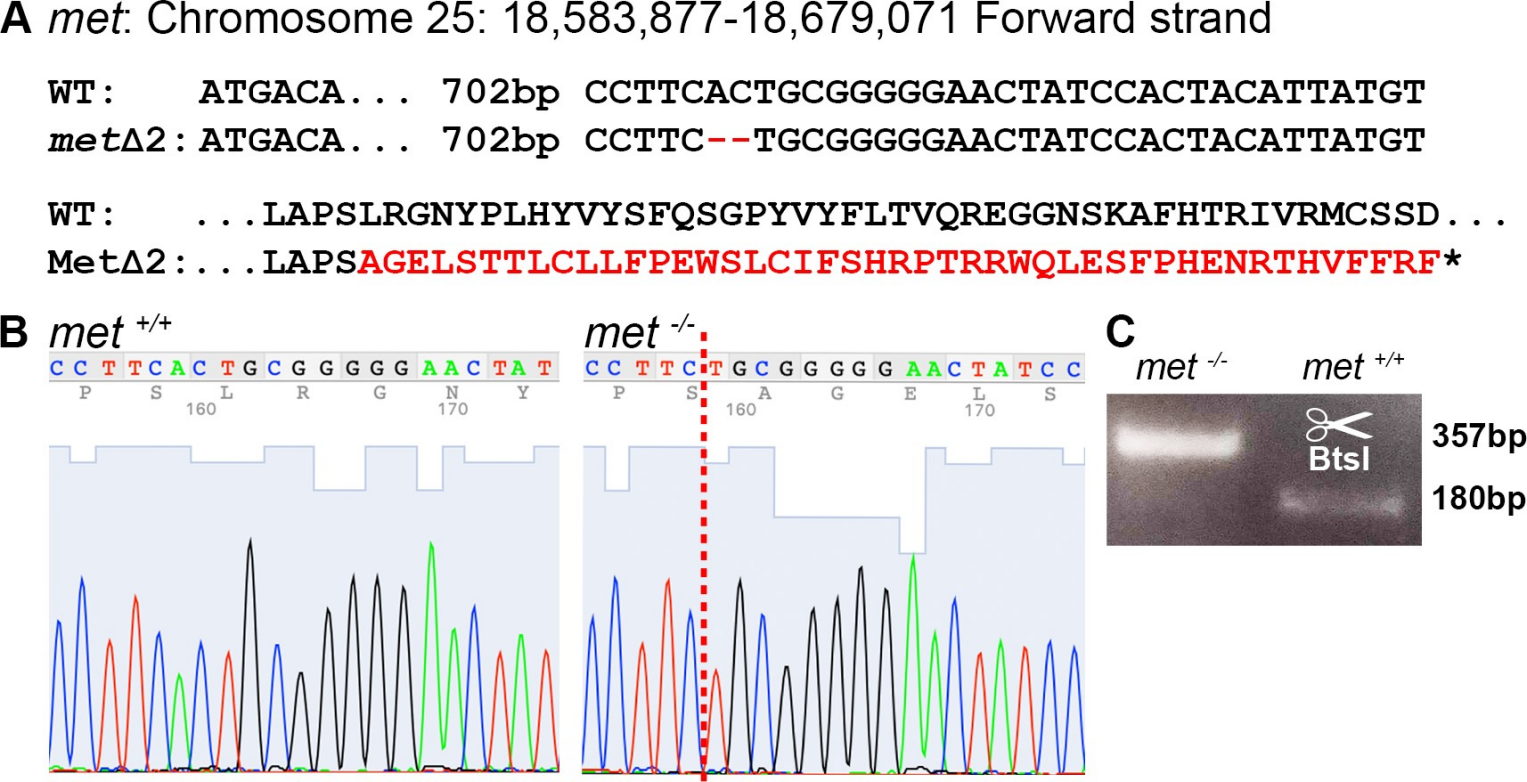
Generation of zebrafish mutants using CRISPR/Cas9. (A) DNA sequence of wt *met* and *metΔ2* (*met*^umu7^) after targeting exon 2 using CRISPR/Cas9 where a 2 bp deletion was identified in *met* which effectively truncated the protein as visualized in amino acid sequence below. Red text indicates faulty amino acid, red lines indicates base deletion, * indicates stop codon. (B) Chromatogram depicting wt (+/+) and mutant (-/-) CRISPR/Cas9 target sequences of the *met* gene, red dotted line indicate site of deletion. (C) Gel electrophoresis representing *met*^*-/-*^ mutant and wt PCR-bands after BtsI digestion (BtsI recognizes 5’-NNCACTGC-3’).

**Fig 2:**
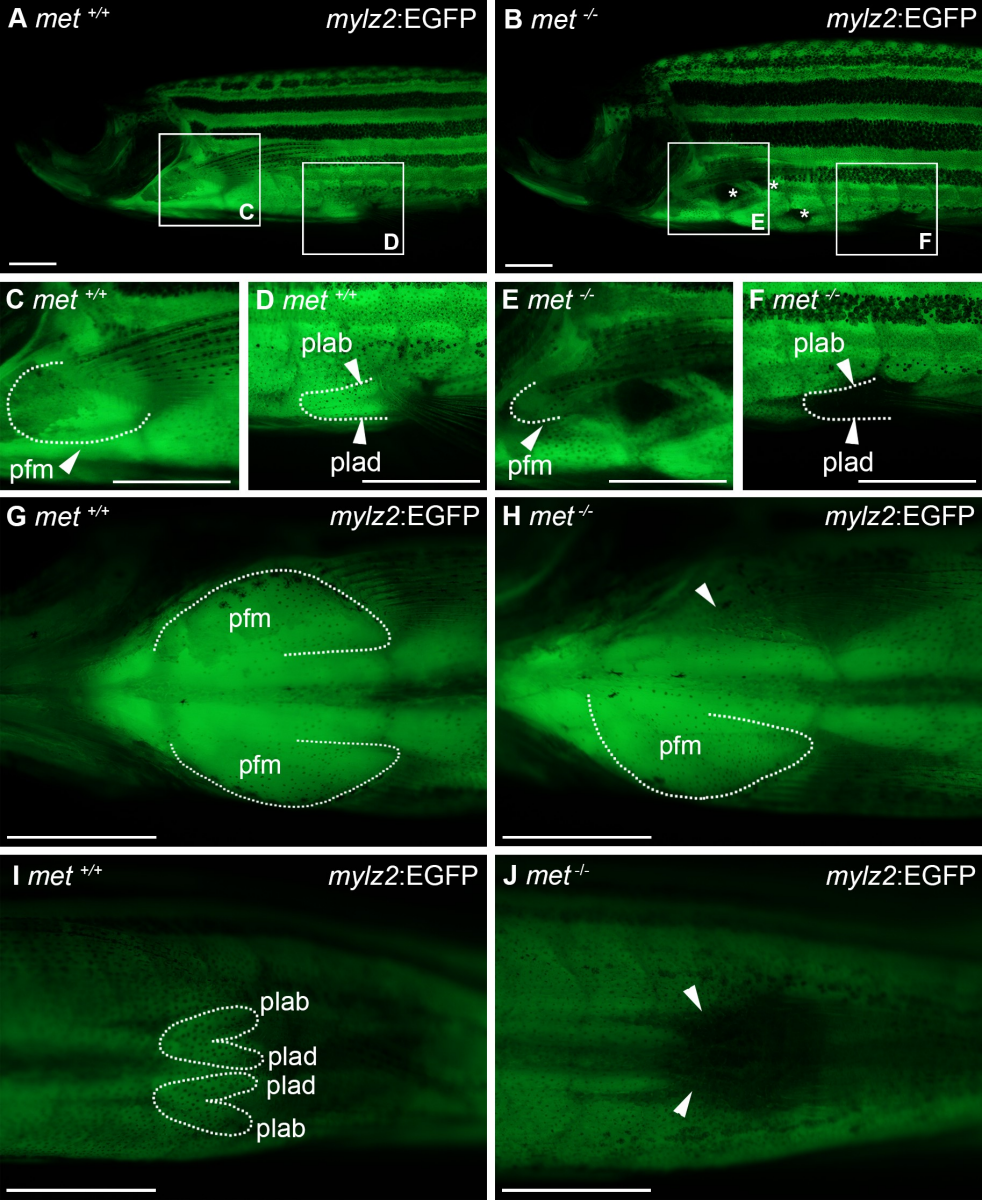
Pectoral and pelvic fin musculature is affected in adult *met*^*-/-*^ mutant zebrafish. Lateral view of *mylz2*:EGFP transgenic expression in pectoral and pelvic fin musculature of (A) *met*^*+/+*^ (n = 18) and (B) *met*^-/-^ (n = 15) adult zebrafish, squares indicate areas of enlargement in C-F. Ventral view of *mylz2*:EGFP transgenic expression in pectoral fin muscle of (G) *met*^*+/+*^ (n = 18) and (H) *met*^-/-^ (n = 15) adult zebrafish. Ventral view of *mylz2*:EGFP transgenic expression in pelvic fin muscle of (I) *met*^*+/+*^ (n = 18) and (J) *met*^-/-^ (n = 15) adult zebrafish. Dashed lines outline muscles as indicated, asterisks indicate areas affected in the hypaxial musculature, arrowheads indicate lack of muscle. Abbreviations: pfm: pectoral fin muscle; plab: abductor pelvicus; plad: adductor pelvicus. Scale bar: 1 mm.

To study the roles of *met* during myogenesis, and particularly in the formation of muscles deriving from MMPs, we generated zebrafish strains carrying mutations in the *met* gene using Crispr/Cas9. Several mutations were identified and versions resulting in frameshifts and early premature stop codons were used to generate F2 generations, which were kept for further analysis (Fig 1). Using whole mount *in situ* hybridisation, we found that *met* expression was reduced in the fin buds of *met*^-/-^ embryos 48 hours post fertilization (hpf), likely due to a combination of non-sense mediated decay and a failure of MMP migration (S1 Fig). *met*^*-/-*^ mutants could easily be identified already at 3 days post fertilization (dpf) due to impaired pectoral fin function (S1 and S2 Movies). Despite the fin movement deficit, *met*^-/-^ mutants survived into adulthood when reared separated from their siblings. To assess the role of *met*, as well as the reason for the pectoral fin impairment in the *met*^-/-^ mutants, we analysed differentiated muscle fibres in the paired appendages by comparing the expression of *mylz2*:EGFP in *met*^*-/-*^ mutants with wildtype (wt) expression in adult zebrafish. As expected, the formation of differentiated muscle fibres within the pectoral fins as well as pelvic fins in *met*^-/-^ mutants was disturbed (Fig 2). Asymmetrical differences were observed in the pectoral fins in 40% of the examined fish (n = 10). In addition, hypaxial muscle was affected in adult *met*^*-/-*^ mutants, resulting in areas with reduced muscle tissue in all of the examined fish (n = 11) (asterisks in Fig 2B). The reduction of hypaxial muscle was however not as severe as in the pectoral and pelvic fin musculature and was occasionally asymmetrical (9% n = 11) (Fig 2).

**Fig 3:**
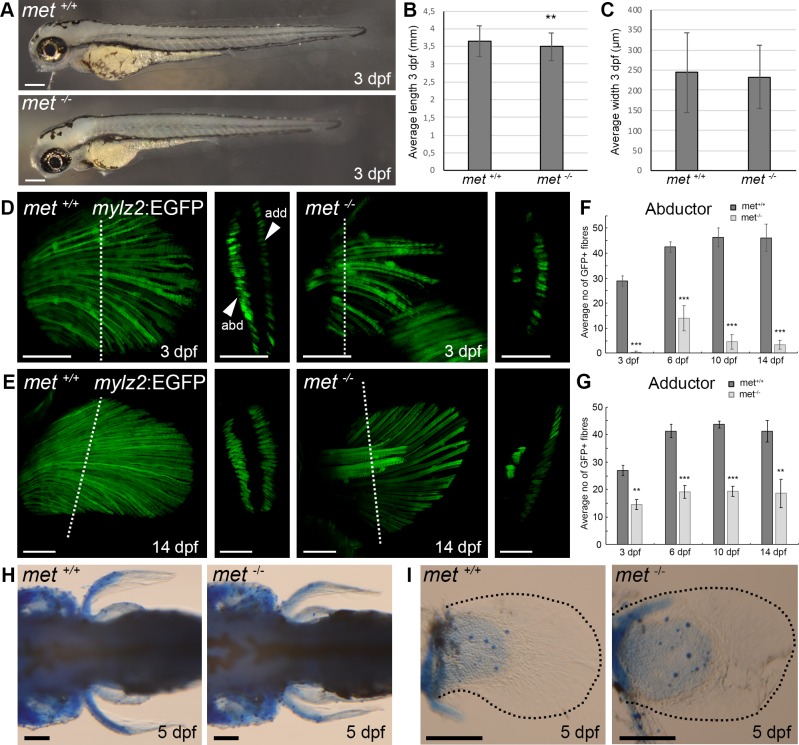
Met mutant embryos display a pectoral fin muscle phenotype. (A) Lateral view of *met*^*+/+*^ and *met*^*-/-*^ embryos at 3 dpf. (B) Average anterior-posterior length of embryo and (C) average myotome dorsal-ventral width at the level of the most posterior part of the yolk extension of *met*^*+/+*^ (n = 6) and *met*^*-/-*^ (n = 5) embryos at 3 dpf. (D) Transgenic expression of *mylz2*:EGFP in the pectoral fin of *met*^*+/+*^ siblings and *met*^*-/-*^ mutant zebrafish embryos at 3 dpf, dashed line indicate area of transverse section presented to the right, which shows the two separate abductor and adductor muscles of the pectoral fin. (E) Transgenic expression of *mylz2*:EGFP in the pectoral fin of *met*^*+/+*^ siblings and *met*^*-/-*^ mutant zebrafish at 14 dpf, dashed line indicate area of transverse section presented to the right, which shows the two separate abductor and adductor muscles of the pectoral fin. Average number of *mylz2*:EGFP^+^ fibers in the pectoral (F) abductor and (G) adductor fin muscle of different *met*^*+/+*^ siblings (dark grey) and *met*^*-/-*^ mutant (light grey) zebrafish at 3 dpf (n = 6 for *met*^*+/+*^ and n = 5 for *met*^*-/-*^), 6 dpf (n = 5 and n = 5), 10 dpf (n = 5 and n = 5) and 14 dpf (n = 6 and n = 5). Error bars indicate S.E.M. Significance was calculated using students t-test where p<0.05 was considered significant, * p<0.05, ** p<0.01, *** p<0.001. (H) Dorsal view and (I) enlargement of pectoral fin of *met*^*+/+*^ siblings (n = 5) and *met*^-/-^ mutant (n = 5) embryos at 5 dpf stained with alcian blue to visualize cartilage, dashed black lines outline pectoral fins. Abbreviations: add: adductor; abd: abductor. Scale bars: A: 200 μm, D-E: 50 μm, H-I:100 μm.

The gross morphology of the *met*^-/-^ mutant embryos was generally normal, even though the embryos were marginally shorter than their wt siblings (Fig 3A–3C, S1 File). However, when we examined the MMP derived structures we found severe developmental defects. The larval pectoral fins are composed of a central disc-like flexible endoskeletal chondroid section flanked by a layer of muscle fibres on each side, the anterior abductor and the posterior adductor muscle [36, 37]. At 5 dpf these muscles consist of an equal number of muscle fibres on both sides of the endoskeletal disc and account for the movement of the pectoral fins and the fine-tuning of locomotion [37]. In the *met*^-/-^ embryos, we observed that the abductor/adductor symmetry was skewed. We found that the number of fibres in the abductor muscle was greatly reduced in all examined *met*^-/-^ embryos at 3 dpf (Fig 3D, 3F and 3G, S1 File). In fact, at 3 dpf the abductor was completely missing in 80% of the analysed *met*^-/-^ embryos. The adductor muscle was however still present in all examined *met*^-/-^ mutants (n = 20), even though the average number of fibres within the adductor was significantly reduced compared to wt siblings (Fig 3D, 3F and 3G). The same mutant muscle anatomy also persisted at 6 dpf, 10 dpf and 14 dfp, indicating that no compensatory mechanism rescues the phenotype (Fig 3E–3G). The endoskeletal disc that separates the abductor and adductor muscle in the pectoral fins formed normally in *met*^-/-^ embryos (Fig 3H and 3I), indicating that *met* is not involved in the formation of the chondroid structures within the fin bud and the developing pectoral fin.

### Involvement of met in non-appendicular MMP derived muscle

**Fig 4:**
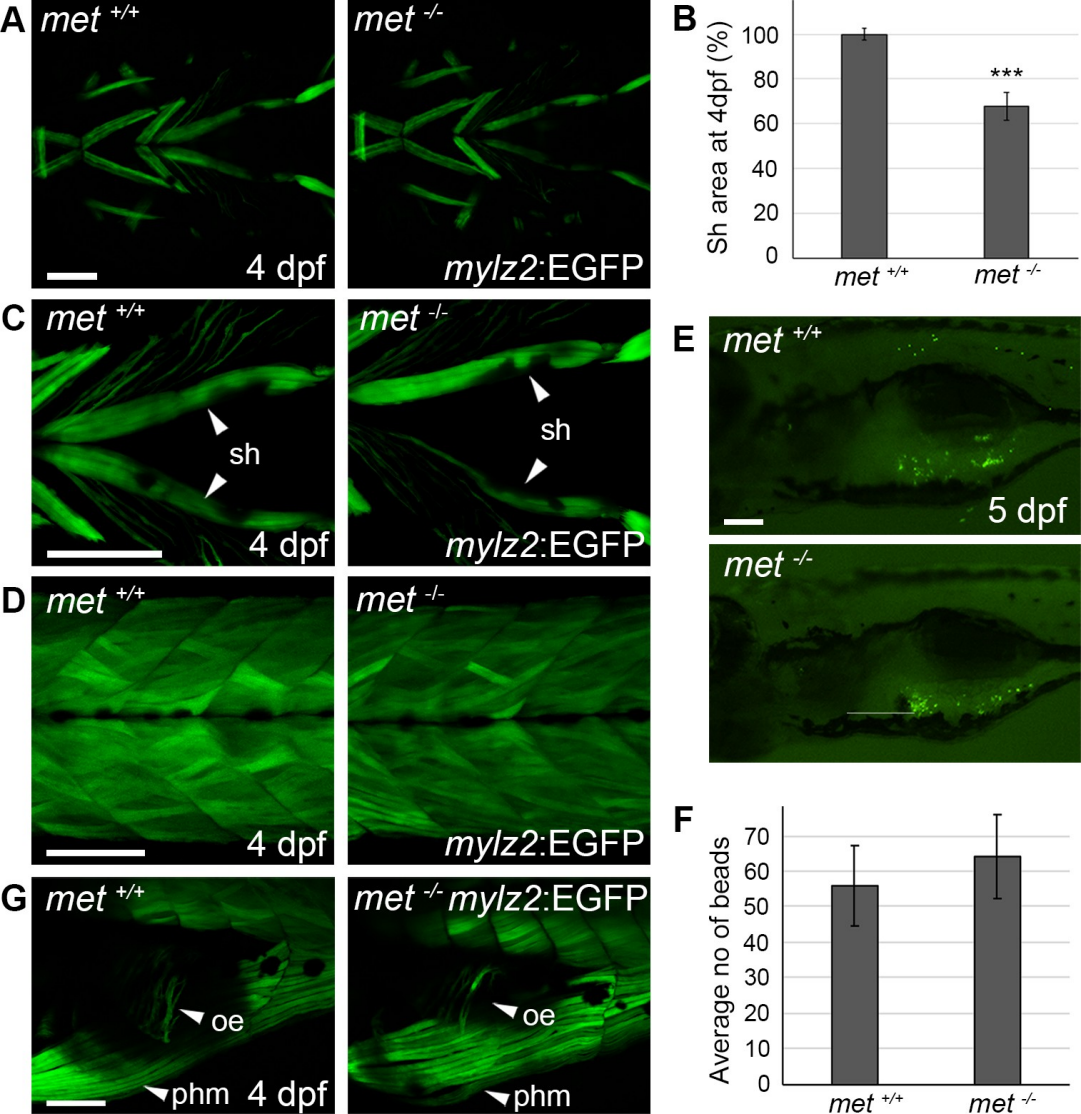
Met is required for proper sternohyoideus formation. (A) Ventral view of transgenic *mylz2*:GFP expression in the craniofacial muscles of *met*^*+/+*^ siblings (n = 5) and *met*^-/-^ mutant (n = 5) embryos at 4 dpf. (B) Average GFP^+^ sternohyoideus area of *met*^-/-^ mutant embryos (n = 6) in proportion to *met*^*+/+*^ siblings (n = 6) at 4 dpf. (C) Ventral view of transgenic *mylz2*:EGFP expression in the sternohyoideus of *met*^*+/+*^ siblings and *met*^-/-^ mutant embryos at 4 dpf. (D) Lateral view of transgenic *mylz2*:GFP expression in the somites of *met*^*+/+*^ siblings (n = 5) and *met*^-/-^ mutant embryos (n = 5) at 4 dpf. (E) Lateral view of *met*^*+/+*^ siblings (n = 10) and *met*^-/-^ mutant larvae (n = 9) fed fluorescent beads at 5 dpf, the average number of beads detected in the stomach is presented in (F). (G) Lateral view of transgenic *mylz2*:EGFP expression in the oesophagus of *met*^*+/+*^ (n = 5) siblings and *met*^-/-^ (n = 5) mutant embryos at 4 dpf. Error bars indicate S.E.M. Significance was calculated using students t-test where p<0.05 was considered significant, * p<0.05, ** p<0.01, *** p<0.001. Abbreviations: oe: oesophagus; phm: posterior hypaxial muscle; sh: sternohyoideus. Scale bar: 100 μm.

The sternohyoideus and posterior hypaxial muscle both expressed *mylz2*:EGFP in *met*^-/-^ embryos at 4 dpf (Fig 4A, 4C and 4D). Both these muscles displayed a relatively normal morphology and anatomical orientation, although the sternohyoideus was thinner and in 50% of the examined embryos asymmetrically affected (>20% muscle area difference, n = 6) (Fig 4A–4C). Other non-MMP derived muscles in the myotome as well as cranial muscle appeared un-affected in the *met*^*-/-*^ embryo (Fig 4A and 4D). Previously it has been described that the oesophagus muscle derives from MMPs [31], we thus analysed if the capability of feeding could be impaired among the *met*^-/-^ zebrafish. We did however not find any significant difference in the amount of ingested fluorescent particles between *met*^-/-^ and wt siblings at 5 dpf (Fig 4E and 4F). The skeletal muscle within the oesophagus also appeared to be normal in the *met*^-/-^ embryo at 4 dpf (Fig 4G) arguing against a role for *met* dependence for the ingestion apparatus.

### Met and Pax3 are expressed in MMP cells

**Fig 5:**
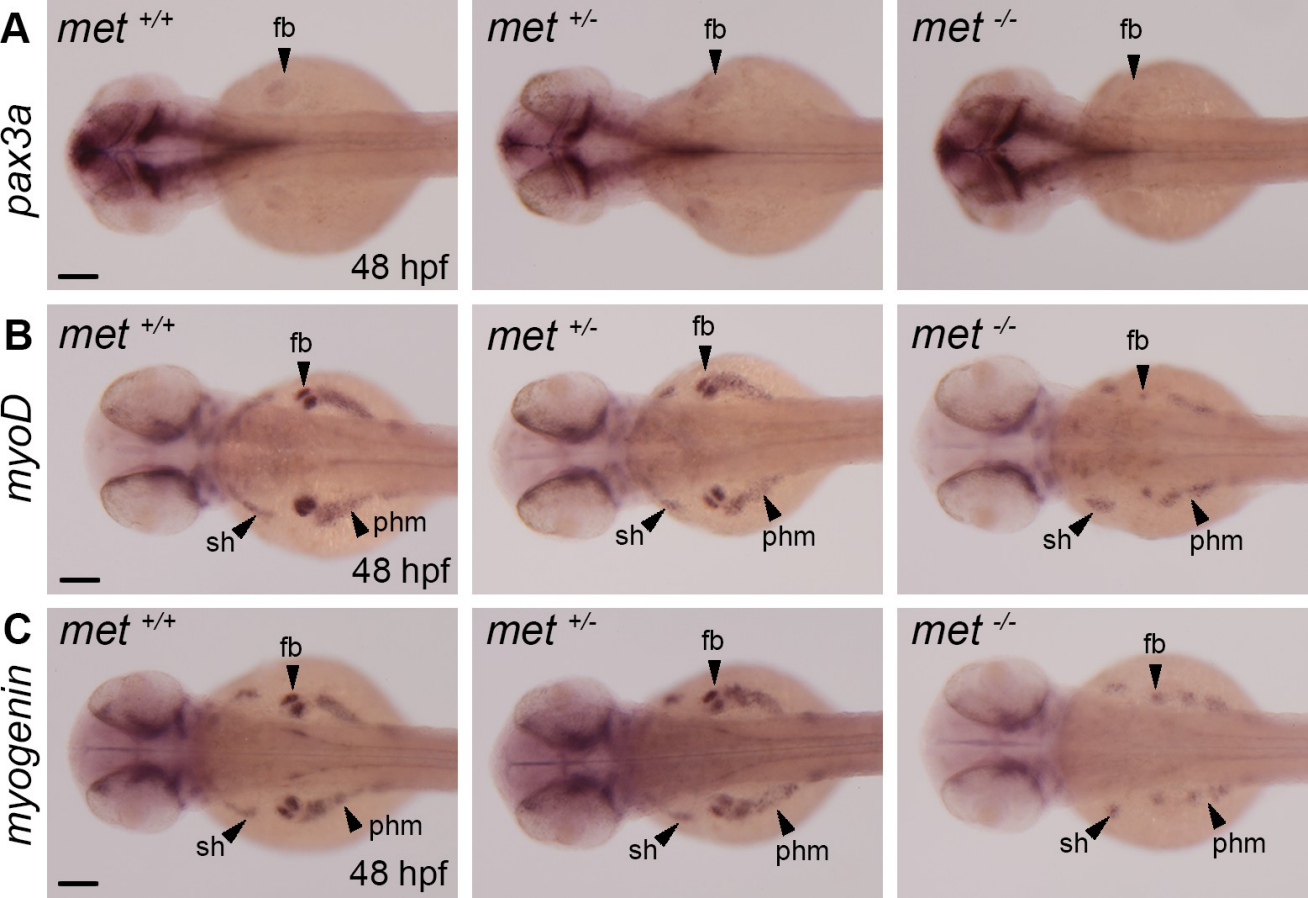
The expression of myogenic markers is severely reduced in MMPs in *met*^*-/-*^ mutant embryos. Whole mount *in situ* hybridization showing the expression of (A) *pax3a* in *met*^*+/+*^ siblings (n = 5), *met*^+/-^ heterozygotes (n = 5) and *met*^*-/-*^ (n = 8) mutant embryos, (B) *myoD* in *met*^*+/+*^ siblings (n = 5), *met*^+/-^ heterozygotes (n = 5) and *met*^*-/-*^ mutant embryos (n = 12) and (C) *myogenin* in *met*^*+/+*^ siblings (n = 5), *met*^+/-^ heterozygotes (n = 5) and *met*^*-/-*^ mutant embryos (n = 10) at 48 hpf. Abbreviations: fb: fin bud; sh: sternohyoideus; phm: posterior hypaxial muscle. Scale bar: 100 μm.

To study the role of *met* in MMP cells, we analysed the embryonic expression of the myogenic markers *pax3a*, *myoD* and *myogenin* in MMPs and their derived tissues and found that the expression of these markers was reduced in all analysed *met*^-/-^ mutant embryos compared to wt as well as heterozygote siblings (Fig 5A–5C). Particularly the pectoral fin buds displayed clear expressional differences of *myoD* and *myogenin* in *met*^-/-^ mutants versus wt siblings where the fin bud cluster appeared much smaller (Fig 5B and 5C). The expression of *myoD* and *myogenin* in the prospective sternohyoideus and hypaxial muscle seemed less affected although the size of cell clusters was reduced (Fig 5B and 5C). The *met*^*-/-*^ mutant embryos did indeed express *pax3a*, *myoD* and *myogenin* in MMPs, but the MMP clusters within the fin bud appeared smaller than in the wt siblings at 48 hpf (Fig 5A–5C). A variation in pectoral fin cluster size among the *met*^*-/-*^ mutant embryos was also observed, in line with the quantification of the differentiated fibres analysed at 3–14 dpf (Fig 3D–3G). The expression of *myoD* and *myogenin* in non-migratory muscle progenitors, in the myotome did not appear to be affected in the *met*^*-/-*^ mutant embryo (S1 Fig).

**Fig 6:**
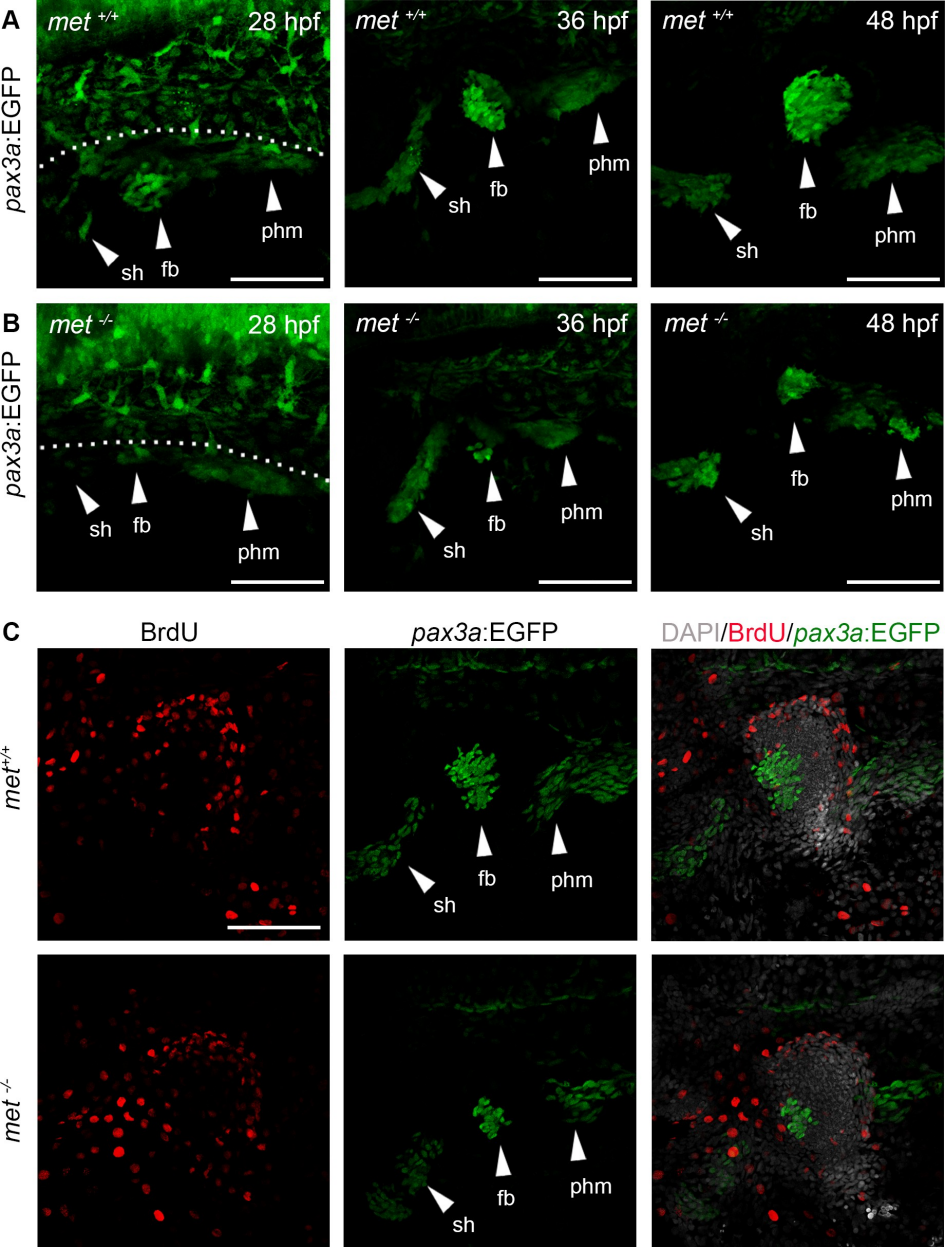
The *pax3a*:EGFP^+^ populations of MMPs migrating out from the somites are reduced in *met*^*-/-*^ mutants. Lateral view of transgenic *pax3a*:EGFP expression in (A) *met*^*+/+*^ siblings and (B) *met*^-/-^ mutant embryos at 28 hpf (n = 6 for *met*^+/+^ and n = 9 for *met*^-/-^), 36 hpf (n = 7 for *met*^+/+^ and n = 5 for *met*^-/-^) and 48 hpf (n = 5 for *met*^+/+^ and n = 5 for *met*^-/-^). (C) *met*^*+/+*^ siblings (n = 5) and *met*^-/-^ mutant (n = 5) *pax3a*:EGFP (green) embryos treated with BrdU (red) from 24 to 48 hpf to visualize proliferating cells. Dashed line in A indicate yolk-somite border. Abbreviations: sh: sternohyoideus; fb: fin bud; phm: posterior hypaxial muscle. Scale bar: 50 μm.

Using *pax3a*:EGFP as a marker, we analysed the MMPs and the formation of sternohyoideus, pectoral fin bud and the prospective hypaxial muscle in *met*^-/-^ mutant embryos. At 28 hpf, the three MMP groups had migrated out from their respective somitic origin to form three identifiable *pax3a*:EGFP expressing cell clusters (Fig 6A and 6B). In wt embryos, these clusters continued to grow as they migrated ventrally and anteriorly (Fig 6A). In the *met*^-/-^ mutants, the fin bud cluster failed to grow and remained visibly smaller than in wt siblings at 36 and 48 hpf (Fig 6B). The differences in sternohyoideus and hypaxial clusters were less evident (Fig 6A and 6B). In order to analyse the migratory event in more detail we performed time-lapse analyses using *pax3a*:EGFP *met*^-/-^ mutants and compared them with wt *pax3a*:EGFP siblings to monitor the formation, growth and migration of these cell clusters from 24 hpf to 72 hpf (S3–S6 Movies), which is when the MMPs are moving out from the anteriormost somites to their positions in the appendicular and distant structures [4, 35]. These analyses indicated that very few cells migrated into the fin bud cluster in the *met*^-/-^ mutant after the initial delamination (S3–S6 Movies). BrdU analyses 24–48 hpf in wt and *met*^-/-^ mutant embryos also revealed that very few cells within the fin bud cluster proliferate (Fig 6C), which indicates that most of the cells in the 48 hpf pectoral fin bud are somite derived.

## Discussion

The genetics underlying limb development in mammals has been thoroughly studied and roles of the genes involved are well known [38]. The pectoral fins in teleost fish are considered homologous to the forelimbs of mammals, but the genetic programmes coordinating the development of these fish limbs are not fully understood. Both in mammals and in zebrafish, somite-derived MMP cells navigate through the lateral mesoderm to inhabit the limb/fin-bud and form the appendicular muscle tissue. In *C-Met*^-/-^ mice, the delamination of MMP cells is disturbed [6], which leads to lack of most MMP-derived appendicular muscle. The MMP cells that will form the pectoral fin musculature become divided into two dorsally and ventrally oriented clusters, which will develop into the abductor and adductor muscle respectively. The mechanism behind this division remains unresolved and seem to differ among different vertebrate groups [30]. We found that pelvic fin muscle was missing in zebrafish *met*^-/-^ mutants and we also observed a drastic reduction of pectoral fin muscle, particularly in the abductor muscle of the pectoral fins (Fig 2 and Fig 3). Similar observations have been made in *Lbx1*^-/-^ mutant mice, where *MyoD* and *Myf5* expression in the dorsal/abductor cluster of the forelimb is reduced to a higher extent than in the ventral/adductor cluster and hindlimb muscle is missing [39, 40]. Lbx1 expression is however not affected by C-met dysregulation and is still expressed in the MMP cells that fail to migrate properly [6]. However, far from all amniote MMP-derived muscle depends on C-met for their migration [30]. For example, the amniote MMP-derived hypobranchial muscle do not require C-met and HGF signalling [41, 42]. A previous study in zebrafish using a morpholino approach to temporary inhibit Met function suggested that many MMP derived muscles were under Met regulation [35]. In our *met*^-/-^ embryos, we did observe altered muscle anatomy outside of the paired appendages, but this observation was limited to marginal reductions, such as in the sternohyoideus (Fig 4A–4C) and in the hypaxial muscle, which also derive from the MMPs [43]. Another MMP-derived muscle in the zebrafish is the oesophagus [31], which appeared to be relatively unaffected in our *met*^*-/-*^ mutants. This indicates that the non-appendicular MMPs still can migrate despite the lack of functional Met and suggests that other molecules may compensate for the dysfunctional *met* in the zebrafish, or alternatively argues for a heterogenous MMP cell pool where only a subset requires *met*. Interestingly, we found that the penetrance of the *met*^*-/-*^ phenotype varied between embryos and occasionally also within the same embryo, where one side could be more severely affected than the other. This suggests that the MMP clusters, by failed migration, occasionally fails to reach a critical size in order to fully form the intended muscle structure. The reason for this variation needs to be studied further, but is likely due to the number of cells that manage to migrate in spite of the lack of Met. We found that the MMP-derived myogenic clusters within the pectoral fin buds have a low proliferation rate after reaching the fin bud (Fig 6C), which indicates that extrinsic signals are unable to compensate for the reduced MMP cell population in the pectoral fin bud by inducing proliferation among the MMP cells. Conclusively, our data provides evidence for an important role for Met in MMP-derived appendicular muscle.

## Materials and methods

### Zebrafish strains and maintenance

Mutant line used was *met*^umu7^ and transgenic lines were *Tg*(*mylz2*:EGFP)^i135^ [44] and *Tg*(*pax3a*:EGFP)^i150^ [45]. Zebrafish were maintained by standard procedures at the Umeå University Zebrafish Facility. All animal experiments were approved by the Umeå djurförsöksetiska nämnd, Dnr: A13-15.

### Generation of met mutant zebrafish using CRISPR/Cas9

*met* zebrafish mutants were generated using methods previously described [46]. In short, gRNA (guide RNA) targeting our gene of interest coupled to a scaffold gRNA was transcribed using the MegaShortScript T7 (Invitrogen) and co-injected with Cas9 protein (New England Biolabs) into one-cell stage zebrafish eggs. Injected embryos were grown to adulthood, outcrossed into wt zebrafish and screened to identify founders containing germline mutations. For *met*, gRNA targeting exon 2 was synthesized using the sequence CCTTCACTGCGGGGGAACTATCC. One frame-shift mutation was identified carrying a 2 bp deletion (Fig 1A). Genotyping was performed using forward 5´-GGGCACTCAGATCCTCAACA and reverse 5´-ATGCACTCAAAGGGCATTTC primers, the product was then digested using the BtsI restriction enzyme (NEB) generating 2 products (180 bp and 177 bp) for wt and no digestion of the product for *met* mutant zebrafish (Fig 1C).

### Whole-mount in situ hybridization

Zebrafish embryos were fixed in 4% paraformaldehyde overnight at desired stages. To prevent pigmentation embryos older than 24 hpf, embryos were reared in 0.003% phenothiourea in embryo medium from 26 ss. Whole-mount *in situ* hybridization was performed as described previously [47] with minor changes; 1% blocking reagent (Roche) was used instead of 2% sheep serum and 2 mg/ml bovine serum albumin. Digoxigenin-labeled RNA probes were detected using 5-bromo-4-chloro-3′-indolyphosphate/nitro blue tetrazolium (Roche). RNA probes were *myoD* (gene bank accession number: NM_131262.2), *myogenin* (NM_131006) and *pax3a* (NM_131277). All comparisons between different expression levels and areas were performed within the same experimental groups.

### Alcian blue staining

To detect cartilage, Alcian blue staining was performed. Zebrafish embryos were fixed in 4% paraformaldehyde overnight and then stored in 100% methanol at −20°C. Embryos were dehydrated and washed in PBT before being transferred to Alcian blue solution (1% concentrated hydrochloric acid, 70% ethanol, 0.1% Alcian blue) and incubated overnight. Embryos were washed in acidic alcohol (5% concentrated hydrochloric acid, 70% ethanol), stepwise rehydrated to H_2_O, and successively cleared in 20% glycerol with 0.25% KOH and 50% glycerol with 0.25% KOH before imaging.

### Fluorescent microsphere swallowing

5 dpf larvae were fed with 0.0026% Fluoresbrite® YG microspheres 1.00 μm (Polysciences, Inc) in embryo medium for 1h, washed extensively and fixed in 4% PFA. Larvae were photographed and the number of fluorescent microspheres in the stomach was counted.

### Brdu treatment

Embryos were dechorionated and incubated in 10 mM 5-Bromo-2′-deoxyuridine (brdU, Sigma) in embryo medium from 26 ss to 48 hpf, fixed in 4% PFA overnight, dehydrated in 100% methanol and stored at −20°C until analysis using immunohistochemistry. For brdU detection, a mouse anti-brdU antibody conjugated to Alexa Fluor 555 was used (1∶20, BD Biosciences), to increase antibody penetrance embryos were treated with 2N HCl and digested with proteinase k before anti-brdU incubation.

### Timelapse

Zebrafish embryos at the desired stage were sedated using tricaine mesylate and mounted in 1% low melt agarose and a z-stack was run every 45 min, after 48 hours the experiment was terminated.

## Supporting information

S1 Movie Movie demonstrating fin movement in *met*^*+/+*^ embryos at 3 dpf. (MP4)

S2 Movie Movie demonstrating fin movement in *met*^*-/-*^ embryos at 3 dpf. (MP4)

S3 Movie Time lapse showing *pax3a*:EGFP^+^ cell movement in wt zebrafish embryos from 26 ss to 48 hpf. (MOV)

S4 Movie Time lapse showing *pax3a*:EGFP^+^ cell movement in wt zebrafish embryos from 48 hpf to 72 hpf. (MOV)

S5 Movie Time lapse showing *pax3a*:EGFP^+^ cell movement in *met*^*-/-*^ mutant zebrafish embryos from 26 ss to 48 hpf. (MOV)

S6 Movie Time lapse showing *pax3a*:EGFP^+^ cell movement in *met*^*-/-*^ mutant zebrafish embryos from 48 hpf to 72 hpf. (MOV)

S1 Fig (A) Dorsal view of whole mount *in situ* showing the expression of *met* in *met*^*+/+*^ siblings (n = 7) and *met*^*-/-*^ (n = 5) mutant embryos at 48 hpf. Scale bar: 100 μm. (B) Lateral view of whole mount *in situ* showing the expression of *myoD* and *myogenin* in *met*^*+/+*^ siblings (n = 7 for *myoD* and 5 for *myogenin*) and *met*^*-/-*^ (n = 5 for *myoD* and 5 for *myogenin*) mutant embryos at 24 hpf. Abbreviation: fb: fin bud. Scale bar: 100 μm.
(TIF)

S1 File Raw data. (XLSX)
