# Supplementary figures and images for: The zebrafish HGF receptor met controls migration of myogenic progenitor cells in appendicular development
Source: PLoS One. 2019 Jul 9;14(7):e0219259. doi: 10.1371/journal.pone.0219259 (PMC6615617; doi:10.1371/journal.pone.0219259)

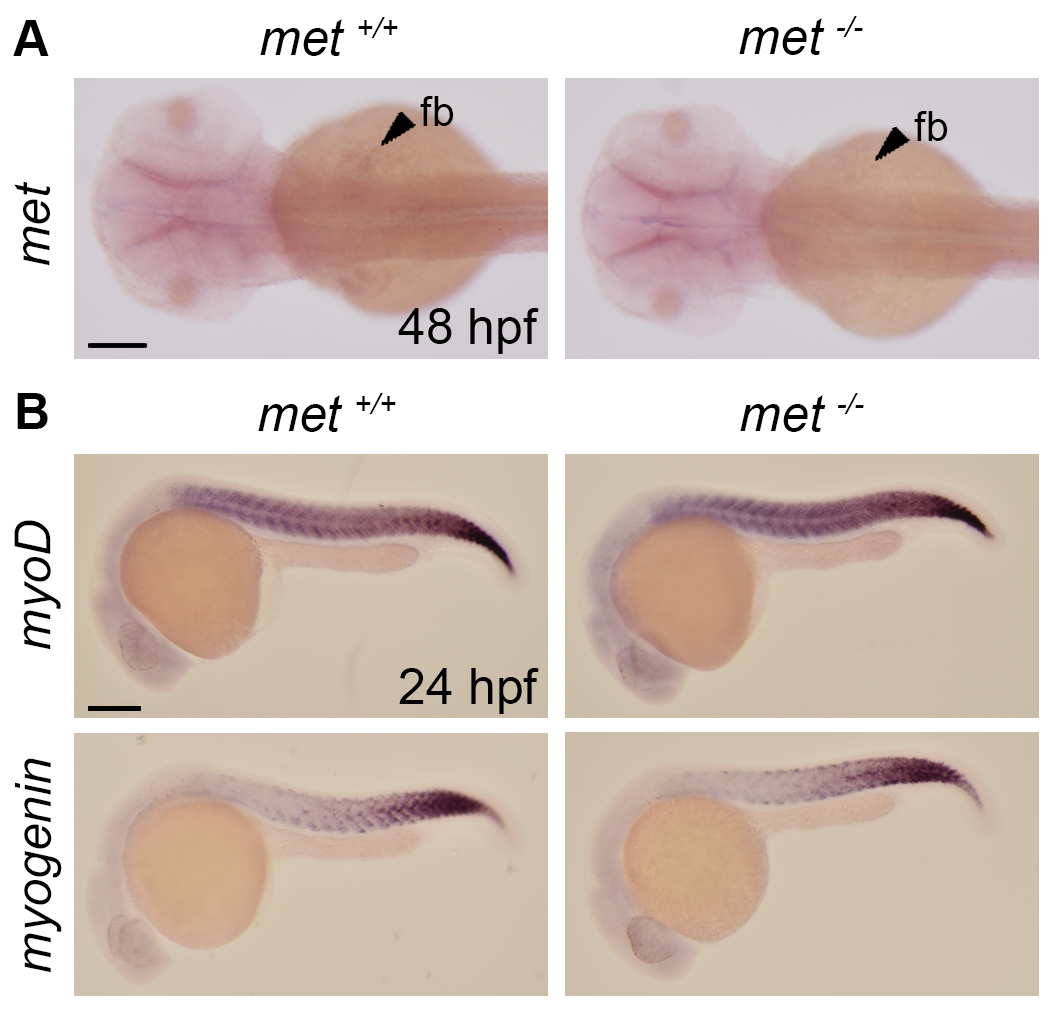

Supplement: S1 Fig — (A) Dorsal view of whole mount in situ showing the expression of met in met+/+ siblings (n = 7) and met-/- (n = 5) mutant embryos at 48 hpf. Scale bar: 100 μm. (B) Lateral view of whole mount in situ showing the expression of myoD and myogenin in met+/+ siblings (n = 7 for myoD and 5 for myogenin) and met-/- (n = 5 for myoD and 5 for myogenin) mutant embryos at 24 hpf. Abbreviation: fb: fin bud. Scale bar: 100 μm. (TIF) [file pone.0219259.s007.tif]
